# Infection in Advanced Chronic Kidney Disease and Subsequent Adverse Outcomes after Dialysis Initiation: A Nationwide Cohort Study

**DOI:** 10.1038/s41598-020-59794-7

**Published:** 2020-02-19

**Authors:** Chih-Hsiang Chang, Pei-Chun Fan, George Kuo, Yu-Sheng Lin, Tsung-Yu Tsai, Su-Wei Chang, Ya-Chung Tian, Cheng-Chia Lee

**Affiliations:** 1 Kidney research center, Department of Nephrology, Chang Gung Memorial Hospital, Chang Gung University, College of Medicine, Taoyuan, Taiwan; 2 Graduate Institute of Clinical Medical Sciences, College of Medicine, Chang Gung University, Taoyuan, Taiwan; 3 Department of Cardiology, Chang Gung Memorial Hospital, Chiayi branch, Chiayi, Taiwan; 4 Clinical Informatics and Medical Statistics Research Center, Chang Gung University, Taoyuan, Taiwan; 5 Division of Allergy, Asthma, and Rheumatology, Department of Pediatrics, Chang Gung Memorial Hospital, Taoyuan, Taiwan

**Keywords:** Preventive medicine, Renal replacement therapy

## Abstract

It remains unclear whether infection events before entering end stage renal disease (ESRD) have a long-term negative impact on patients with advanced chronic kidney disease (CKD) who survive to permanent dialysis. We enrolled 62,872 patients with advanced CKD who transitioned to maintenance dialysis between January 1, 2004 and December 31, 2013. We used multivariable Cox as well as Fine and Gray models to determine the association of pre-dialysis infection exposure with all-cause mortality after starting dialysis. Compared with no infection during advanced CKD, the presence of infection exposure during that period was independently associated with a higher risk of all-cause mortality in the first year of dialysis (hazard ratio [HR] 1.34, 95% confidence interval [CI] 1.27–1.42) and also during the entire follow-up period (HR 1.19, 95% CI 1.16–1.22). The increased risks of all-cause mortality increased incrementally with higher annual number of infections during advanced CKD. Similar results were found for all other adverse outcomes, e.g. post-ESRD infection-related hospitalization and major cardiac and cerebrovascular events. In conclusion, infection events during advanced CKD was associated with increased risks of adverse outcomes after dialysis has been started. Timely interventions in such a vulnerable group may help attenuate these risks.

## Introduction

Advanced chronic kidney disease (CKD) is associated with a marked increase in the risk of all-cause mortality and morbidity, frequently necessitating hospitalization^1,2^. Other than cardiovascular causes, approximately 50% of mortality in patients with CKD can be attributed to non-cardiovascular causes; infection is now increasingly recognized as one of these causes, particularly as some such infection events may be preventable^2–5^. Infection is also a common cause of hospitalization in patients with CKD and studies have shown a strong graded association between the incidence of infection and reduced kidney function^6–9^.

Infection events in patients receiving dialysis not only contributes to a higher risk of death but is also associated with substantial morbidity. For example, research has reported that 15.3% of patients with infection-related hospitalization (IRH) required an intensive care unit stay while 28.6% required prolonged hospitalization^10^ and 21%–34% were readmitted within 30 days^11–13^, and even that IRH increased the risk of subsequent cardiovascular events^14^, cumulatively constituting a substantial burden on the health care system. Hence, identifying dialysis patients at risk of IRH is of paramount importance.

Few studies have addressed the consequences of an infection event in non-dialysis patients with advanced CKD. A recent analysis of the Canadian prospective cohort (CanPREDDICT) comprising 2,370 patients with advanced CKD showed that an infection episode was independently associated with an increased risk of end-stage renal disease (ESRD) or other adverse outcome before dialysis, such as cardiovascular events and death^15^. However, it remains unclear whether infection events before entering ESRD have a long-term negative impact on patients with advanced CKD who survive to permanent dialysis. To address this knowledge gap, we investigated the association of infection exposure during advanced CKD and adverse outcomes, e.g. all-cause mortality, recurrent IRH, major cardiac and cerebrovascular events (MACCE), after dialysis initiation by using a nationwide representative population with advanced CKD transitioning to ESRD. We hypothesized that infection exposure in advanced CKD might not only lead to a higher risk of adverse outcomes before ESRD but also have a persistently negative impact after transitioning to permanent dialysis.

## Results

### Study population characteristics

**Figure 1:**
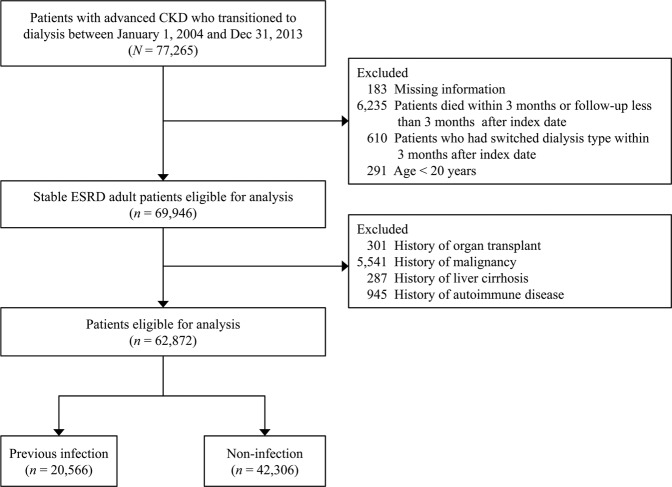
Flowchart of patient inclusion.

The study population selection process is shown in Fig. 1. We identified 77,265 patients with advanced CKD who started permanent dialysis (either peritoneal dialysis or hemodialysis) between January 1, 2004 and December 31, 2013. We excluded patients who switched dialysis type within 3 months of starting dialysis (n = 610) in order to investigate the impact of different dialysis modalities. Patients were also excluded if they had a history of organ transplant, malignancy, liver cirrhosis, or autoimmune disease because they represented a substantially different population with varying susceptibility to infection than our target population. Ultimately, a study population of 62,872 patients with advanced CKD who entered permanent dialysis were eligible for analysis.

Table 1 shows the baseline characteristics of the study population, stratified by infection exposure status during pre-dialysis advanced CKD. The mean age was 63.1 ± 13.7 years, 51.6% of patients were male, and 58.8% of patients had diabetes. Of the patients, 20,566 (32.7%) had at least one infection episode whereas 42,306 (67.3%) had no infection. Compared with the non-infection group, the infection group was older, was more likely to be female, and had a higher prevalence of diabetes, ischemic heart disease, history of heart failure and myocardial infarction, prior cerebrovascular disease, hypoalbuminemia, and dementia at baseline. The follow-up duration was shorter in the patients with infection than in those without infection (3.2 vs. 3.8 years, P < 0.001). Other detailed baseline characteristics including some of comorbidities and 14 medications between two groups are available in Supplementary Table S1.

**Table 1:** Selected baseline characteristics of patients stratified by infection exposure status during pre-dialysis advanced CKD.

Variable	All (n = 62,872)	Infection (n = 20,566)	Non-infection (n = 42,306)	P value
**Demographic**				
Age (y)	63.1 ± 13.7	65.6 ± 13.6	61.9 ± 13.6	< 0.001
Female	30,404 (48.4)	11,322 (55.1)	19,082 (45.1)	
No. of nephrologist outpatient visits in the previous year	11.7 ± 9.1	12.1 ± 9.0	11.5 ± 9.1	< 0.001
**Modality**				< 0.001
Hemodialysis	54,291 (86.4)	18,206 (88.5)	36,085 (85.3)	
Peritoneal dialysis	8,581 (13.6)	2,360 (11.5)	6,221 (14.7)	
**Initial dialysis access type for hemodialysis**				< 0.001
Fistula	34,651 (63.8)	9,428 (51.8)	25,223 (69.9)	
Graft	6,155 (11.3)	2,602 (14.3)	3,553 (9.8)	
Tunneled-catheter	13,485 (24.8)	6,176 (33.9)	7,309 (20.3)	
**Comorbidity**				
Hypertension	56,987 (90.6)	18,699 (90.9)	38,288 (90.5)	0.090
Diabetes mellitus	36,952 (58.8)	12,489 (60.7)	24,463 (57.8)	< 0.001
Ischemic heart disease	16,908 (26.9)	5,964 (29.0)	10,944 (25.9)	< 0.001
Dementia	1,924 (3.1)	901 (4.4)	1,023 (2.4)	< 0.001
History of heart failure	15,675 (24.9)	6,209 (30.2)	9,466 (22.4)	< 0.001
Previous ischemic stroke	10,315 (16.4)	4,056 (19.7)	6,259 (14.8)	< 0.001
Old myocardial infarction	4,189 (6.7)	1,658 (8.1)	2,531 (6.0)	< 0.001
Hypoalbuminemia	2,794 (4.4)	1,754 (8.5)	1,040 (2.5)	< 0.001
Follow-up duration (y)	3.6 ± 2.6	3.2 ± 2.4	3.8 ± 2.6	< 0.001

Data are presented as frequency (percentage) or mean ± standard deviation.

**Figure 2:**
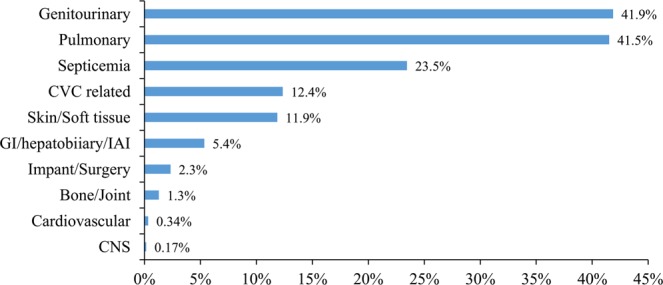
Types of infection during advanced CKD. Abbreviations: CVC, central venous catheter; GI, gastrointestinal; IAI, intra-abdominal infection; CNS, central nervous system.

Figure 2 exhibited the nature of the infectious events during advanced CKD. Of the 20,566 patients with prior infection, the most frequent infections were urinary tract infection (41.9%) and pulmonary infection (41.5%), followed by septicemia (23.5%), central venous catheter-related infection (12.4%) and soft tissue infections (11.9%). The average time and standard deviation in years from an infection event during advanced CKD to ESRD, post-ESRD IRH, post-ESRD MACCE, and all-cause mortality were 0.4 ± 0.8, 1.7 ± 1.8, 2.5 ± 2.1, and 3.0 ± 2.3, respectively (data not shown).

### Association of infection exposure during advanced CKD with post-ESRD all-cause mortality and IRH

There were 25,475 (40.5%) deaths during a mean follow-up of 3.6 years (standard deviation: 2.4 years) following dialysis initiation. As shown in Table 2, the infection and non-infection groups had 9,556 (46.5%) deaths and 15,919 (37.6%) deaths, respectively. After the covariates were controlled for, the presence of infection exposure during advanced CKD was independently associated with a higher risk of all-cause mortality (hazard ratio [HR] 1.19, 95% CI 1.16–1.22) compared with the absence of any infection episodes. This increased risk was higher in the first year after starting dialysis (HR 1.34, 95% CI 1.27–1.42) than later during follow-up.

**Table 2:** Follow-up outcomes of primary interest in patients with and without infection history during pre-dialysis advanced CKD.

Outcome	No. of events (%)			Infection vs. non-infection^b^			
	All	Infection	Non-infection	Univariate		Multivariable^c^	
	(n = 62,872)	(n = 20,566)	(n = 42,306)	HR (95% CI)	P value	HR (95% CI)	P value
**1-year follow-up**							
All-cause mortality	5,637 (9.0)	2,681 (13.0)	2,956 (7.0)	1.94 (1.84, 2.04)	<0.001	1.34 (1.27, 1.42)	<0.001
IRH	17,342 (27.6)	7,645 (37.2)	9,697 (22.9)	1.80 (1.75, 1.86)	<0.001	1.44 (1.39, 1.48)	<0.001
Infection death	3,016 (4.8)	1,629 (7.9)	1,387 (3.3)	2.48 (2.31, 2.67)	<0.001	1.58 (1.46, 1.70)	<0.001
MACCE^a^	6,444 (10.2)	2,680 (13.0)	3,764 (8.9)	1.50 (1.43, 1.57)	<0.001	1.17 (1.11, 1.23)	<0.001
**At the end of follow-up**							
All-cause mortality	25,475 (40.5)	9,556 (46.5)	15,919 (37.6)	1.49 (1.46, 1.53)	<0.001	1.19 (1.16, 1.22)	<0.001
IRH	34,711 (55.2)	12,795 (62.2)	21,916 (51.8)	1.48 (1.44, 1.51)	<0.001	1.28 (1.25, 1.31)	<0.001
Infection death	11,766 (18.7)	4,834 (23.5)	6,932 (16.4)	1.60 (1.54, 1.66)	<0.001	1.26 (1.21, 1.31)	<0.001
MACCE^a^	20,472 (32.6)	7,245 (35.2)	13,227 (31.3)	1.23 (1.19, 1.26)	<0.001	1.07 (1.03, 1.10)	<0.001

Abbreviations: CI, confidence interval; CKD, chronic kidney disease; HR, hazard ratio; IRH, infection-related hospitalization; MACCE, major adverse cardiac and cerebrovascular event.

^a^Any patient with acute myocardial infarction, acute ischemic stroke, intracerebral hemorrhage, heart failure, or cardiovascular death.

^b^Except for all-cause mortality, the outcomes were estimated using a subdistribution hazard model that considered all-cause mortality a competing risk.

^c^Adjusted for the variables listed in Supplementary Table S1 except primary renal disease; the follow-up duration was replaced with the index year.

**Figure 3:**
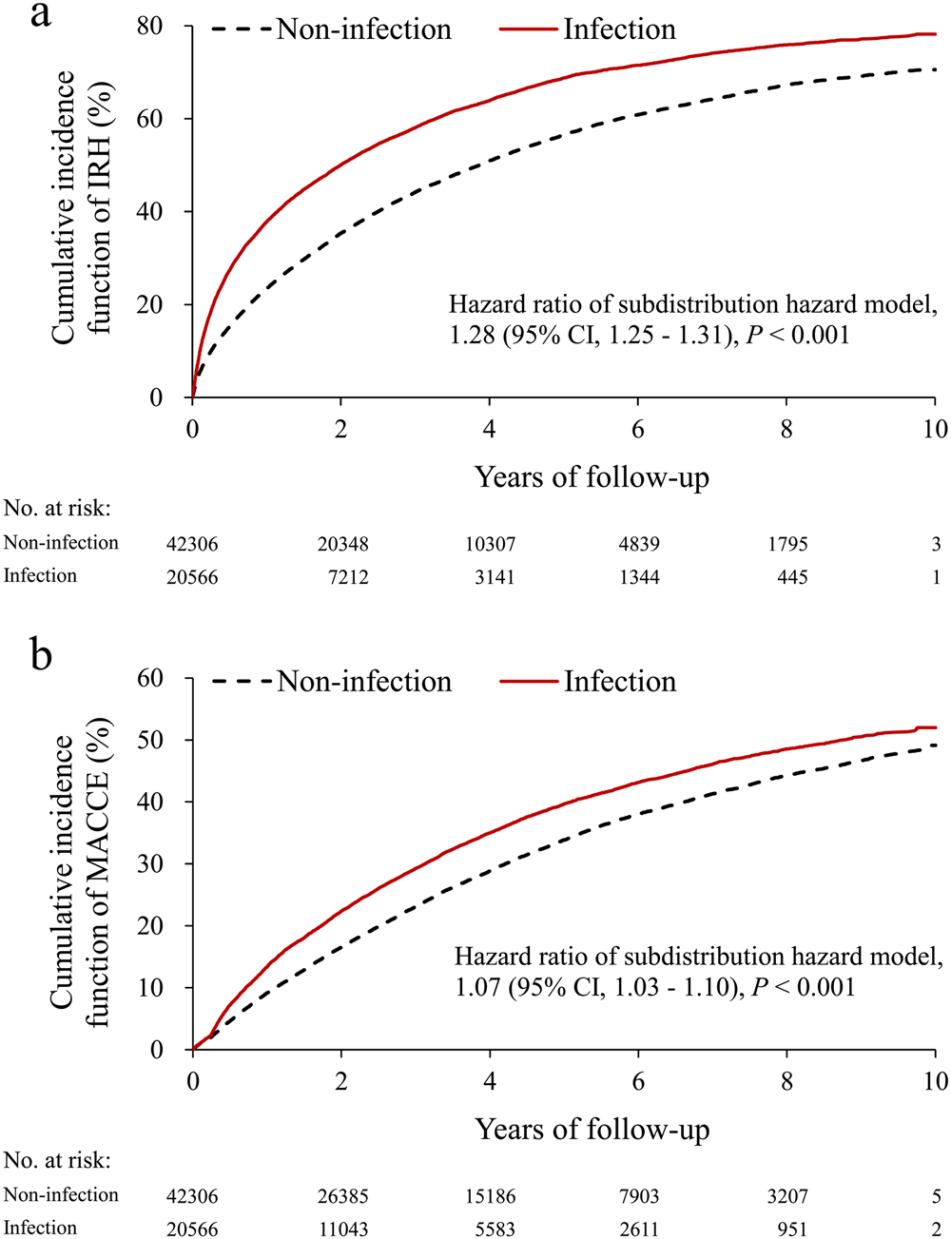
Cumulative incidence of IRH (**a**) and MACCE (**b**) in patients with and without infection during pre-dialysis advanced CKD. Abbreviations: CKD, chronic kidney disease; IRH, infection-related hospitalization; MACCE, major adverse cardiac and cerebrovascular event.

After starting dialysis, 34,711 (55.2%) patients experienced at least one IRH during the entire follow-up, with a crude incidence rate of 22.9 events per 100 person-years. Among the patients who experienced IRH, 17,342 (50%) of them had it during their first year of dialysis. The infection group had significantly higher cumulative incidences (subdistribution HR [SHR] 1.28, 95% CI 1.25–1.31, Fig. 3a) of post-ESRD IRH than the non-infection group did. Similarly, the increased risk of post-ESRD IRH was higher in the first year of dialysis therapy (SHR 1.44, 95% CI 1.39–1.48) than later during follow-up. Notably, the risk of infection-related death was also significantly higher in the infection group than in the non-infection group (HR 1.26, 95% CI 1.21–1.31).

Furthermore, to test the robustness of our results, we categorized the study population with at least one infection episode (infection group, N = 20,566) into four quartiles based on annual number of infections during pre-dialysis advanced CKD. As shown in Table 3, the rate of all-cause mortality increased with the frequency of pre-ESRD infection exposure (P for trend < 0.001), regardless of whether the follow-up was restricted to the first year or the entire follow-up period. The annual number of previous infections during advanced CKD was also incrementally associated with the cumulative incidence of post-ESRD IRH, with significantly higher post-ESRD IRH risks correlated with higher numbers of previous infection events (P for trend < 0.001, Table 3 and Supplementary Fig. S1a).

**Table 3:** Follow-up outcomes of primary interest stratified by annual number of infections during pre-dialysis advanced CKD.

Outcome	No. of events (%), categorized by annual number of previous infections				P trend	P trend^c^
	Group 1 (n = 5,142)	Group 2 (n = 5,154)	Group 3 (n = 5,122)	Group 4 (n = 5,148)		
**1-year follow-up**						
All-cause mortality	396 (7.7)	554 (10.7)	778 (15.2)	953 (18.5)	<0.001	<0.001
IRH^b^	1,374 (26.7)	1,793 (34.8)	2,106 (41.1)	2,372 (46.1)	<0.001	<0.001
Infection death^b^	222 (4.3)	305 (5.9)	483 (9.4)	619 (12.0)	<0.001	<0.001
MACCE^a,b^	435 (8.5)	635 (12.3)	752 (14.7)	858 (16.7)	<0.001	<0.001
**At the end of follow-up**						
All-cause mortality	1,930 (37.5)	2,306 (44.7)	2,514 (49.1)	2,806 (54.5)	<0.001	<0.001
IRH^b^	2,789 (54.2)	3,100 (60.1)	3,380 (66.0)	3,526 (68.5)	<0.001	<0.001
Infection death^b^	950 (18.5)	1,088 (21.1)	1,308 (25.5)	1,488 (28.9)	<0.001	<0.001
MACCE^a,b^	1,465 (28.5)	1,857 (36.0)	1,864 (36.4)	2,059 (40.0)	<0.001	0.003

Abbreviations: CKD, chronic kidney disease; IRH, infection-related hospitalization; MACCE, major adverse cardiac and cerebrovascular event.

The median number of previous infection events was 0.7, 1.8, 4.1, and 12.2 in groups 1, 2, 3, and 4, respectively.

^a^Any patient with acute myocardial infarction, acute ischemic stroke, intracerebral hemorrhage, heart failure, or cardiovascular death.

^b^Except for all-cause mortality, the outcomes were estimated using a subdistribution hazard model that considered all-cause mortality a competing risk.

^c^Adjusted for the variables listed in Supplementary Table S1 except primary renal disease; the follow-up duration was replaced with the index year.

**Figure 4:**
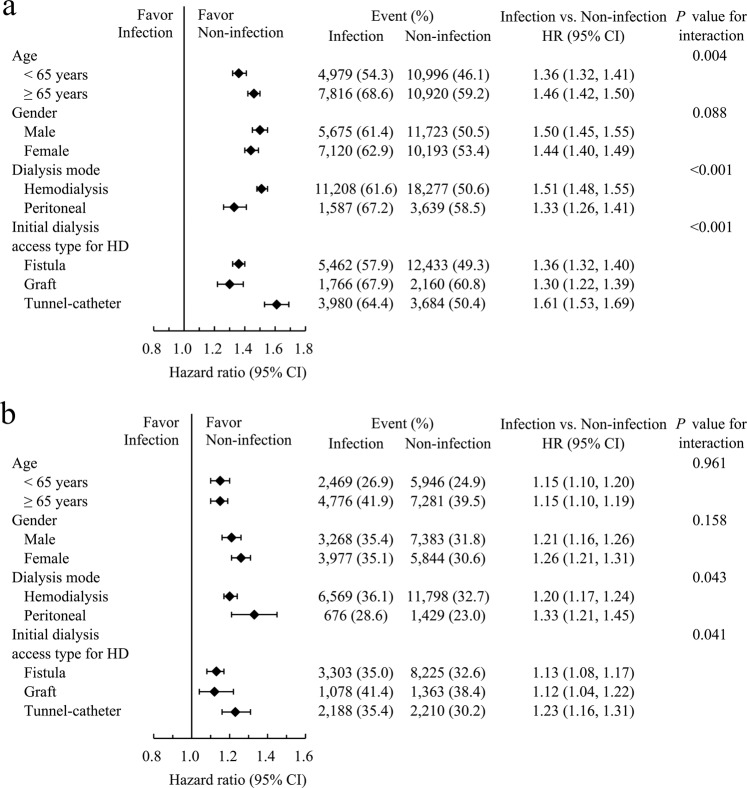
Pre-specified subgroup analysis comparing patients with and without infection during pre-dialysis advanced CKD with IRH (**a**) and MACCE (**b**). Abbreviations: CKD, chronic kidney disease; IRH, infection-related hospitalization; MACCE, major adverse cardiac and cerebrovascular event.

In subgroup analyses, the pattern of associations between pre-ESRD infection exposure and post-ESRD IRH was generally consistent across selected subgroups. However, age and dialysis modalities significantly modified this association: the effect of pre-ESRD infection exposure on the risk of post-ESRD IRH was significantly greater among the older patients and those with hemodialysis, particularly among patients with tunneled-catheter as their initial dialysis access (Fig. 4a).

### Association of infection exposure during advanced CKD with post-ESRD MACCE

During follow-up, 20,472 (32.6%) patients experienced MACCE, including 13,072 cardiovascular deaths. The presence of pre-ESRD infection exposure was associated with a slightly higher risk of MACCE compared with the absence of any infection episodes (SHR 1.07; 95% CI 1.03–1.10, Table 2 and Fig. 3b). However, as the number of previous infections during advanced CKD increased, there was still a stepwise increase in the incidence of MACCE during both the first year (P for trend < 0.001, Table 3) and the entire follow-up period (P for trend = 0.003, Table 3 and Supplementary Fig. S1b). In subgroup analyses, the association between increased risk of MACCE and infection exposure remained consistent across different age and sex groups (Fig. 4b).

### Infections in advanced CKD start a vicious cycle, leading a higher risk of post-ESRD IRH

As shown in Table 4, multivariate logistic regression analysis, after adjustment for related variables, revealed that the presence of pre-ESRD infection exposure was independently associated with a higher risk of post-ESRD IRH in both patients on hemodialysis (SHR 1.35, 95% CI 1.32–1.38) and peritoneal dialysis (SHR 1.30, 95% CI 1.23–1.38). Other factors associated with an at least 20% increased risk of post-ESRD IRH for both dialysis types included older age, hypoalbuminemia, diabetes, chronic obstructive pulmonary disease, prior cerebrovascular disease, and dementia. Notably, a higher number of cumulative nephrology visits during advanced CKD was independently associated with a lower risk of IRH for both dialysis types after starting dialysis.

**Table 4:** Associations between baseline characteristics at dialysis initiation and infection-related hospitalization during follow-up.

Variable	Patients with hemodialysis		Patients with peritoneal dialysis	
	HR (95% CI)	P value	HR (95% CI)	P value
Infection during pre-dialysis advanced CKD	1.35 (1.32, 1.38)	<0.001	1.30 (1.23, 1.38)	<0.001
Age (per 10 y)	1.29 (1.27, 1.30)	<0.001	1.21 (1.18, 1.24)	<0.001
Female	0.99 (0.97, 1.01)	0.412	1.04 (0.98, 1.09)	0.227
CKD duration (per 1 y)	0.998 (0.995, 1.002)	0.373	0.995 (0.987, 1.003)	0.197
No. of prior nephrologist outpatient visits	0.987 (0.986, 0.989)	<0.001	0.994 (0.991, 0.997)	<0.001
**Dialysis access**				
Fistula	1 (Reference)	—	—	—
Graft	1.28 (1.24, 1.32)	<0.001	—	—
Tunneled-catheter	1.40 (1.36, 1.44)	<0.001	—	—
Hypoalbuminemia	1.35 (1.28, 1.42)	<0.001	1.27 (1.09, 1.47)	0.002
Polycystic kidney disease	1.05 (0.97, 1.14)	0.251	1.25 (1.03, 1.51)	0.025
Hypertension	0.97 (0.93, 1.01)	0.127	0.94 (0.86, 1.03)	0.185
Diabetes mellitus	1.42 (1.38, 1.46)	<0.001	1.52 (1.43, 1.61)	<0.001
Chronic obstructive pulmonary disease	1.29 (1.24, 1.34)	<0.001	1.22 (1.06, 1.41)	0.007
Peripheral arterial disease	1.18 (1.11, 1.24)	<0.001	0.99 (0.82, 1.19)	0.902
Ischemic heart disease	1.03 (0.99, 1.06)	0.060	1.13 (1.05, 1.22)	0.002
Dementia	1.41 (1.33, 1.49)	<0.001	1.55 (1.24, 1.92)	<0.001
History of heart failure	1.21 (1.17, 1.24)	<0.001	1.14 (1.05, 1.24)	0.001
Previous ischemic stroke	1.26 (1.23, 1.30)	<0.001	1.32 (1.21, 1.45)	<0.001
Previous hemorrhage stroke	1.28 (1.19, 1.37)	<0.001	1.24 (1.01, 1.51)	0.039
Old myocardial infarction	1.11 (1.06, 1.17)	<0.001	0.95 (0.82, 1.09)	0.440

CI, confidence interval; CKD, chronic kidney disease; HR, hazard ratio.

### Sensitivity analysis

The sensitivity analysis yielded similar results to that of the primary analysis by excluding patients with AKI during advanced CKD period that potentially had rapid decline of renal function (Supplementary Table S2).

## Discussion

This national population-based cohort study of 62,872 patients with advanced CKD revealed that approximately one-third of the patients experienced at least one infection event before transitioning to permanent dialysis. As hypothesized, we found that pre-ESRD infection exposure was associated with higher risks of all-cause mortality, IRH, and MACCE after dialysis initiation. These increased risks were higher in the first year but remained significant throughout the entire follow-up period. These results are strengthened by a dose-dependent relationship observed between annual number of pre-ESRD infection episodes and post-ESRD adverse outcomes, suggesting that infections in advanced CKD start a vicious cycle.

Accumulating evidence indicates that infection is a frequent event in patients with non-dialysis advanced CKD and that reduced estimated glomerular filtration rate (eGFR) is associated with a higher risk of subsequent short-term mortality. In a study of 25,675 participants from a single Canadian health region, individuals with eGFRs of less than 30 mL/min/1.73 m^2^ were found to have a 3.5-fold higher risk of bloodstream infection and more than 4-fold higher risk of subsequent 30-day mortality compared with those with eGFRs of 60 mL/min/1.73 m^2^ or higher^6^. A recent analysis of the Atherosclerosis Risk in Communities cohort also demonstrated that participants with eGFRs of 15–29 mL/min/1.73 m^2^ showed an approximately 3.5-fold higher risk of infection compared with those with eGFRs of 90 mL/min/1.73 m^2^ or more^9^. Moreover, recurrent IRH was more frequent among participants with lower eGFRs (8.9%, 11.5%, 22.5%, and 42.1% in those with eGFRs ≥ 90, 60–89, 30–59, and 15–29 mL/min/1.73 m^2^, respectively). Little is known about the long-term negative impact of infection events or recurrent infections among advanced CKD patients who survive to permanent dialysis, particularly among Asians, who appear to have higher rates of entering permanent dialysis^16^. Herein, we provide further evidence of an association between infection exposure during advanced CKD and post-ESRD adverse outcomes irrespective of all-cause mortality, IRH, or MACCE.

Our finding regarding the substantial burden of infection in dialysis patients is consistent with the results of a previous analysis of 333,453 incident dialysis patients from the U.S. Renal Data System, which showed a 31% cumulative incidence rate of IRH in the first year and 52.5% at 3 years^17^. Therefore, early identification of patients with a higher risk of IRH would likely minimize the overall health care expenditure on dialysis patients, as each IRH can spiral into a vicious cycle resulting in substantial adverse consequences, including worsening nutritional status^18^, readmission^11–13^, and cardiovascular event^14^. Similar to previous studies, we found that older age, diabetes, heart failure, chronic obstructive pulmonary disease, and prior cerebrovascular disease are risk factors for IRH in incident dialysis patients irrespective of their initial dialysis modality^2,19,20^. The finding that catheter as initial vascular access was associated with a higher risk of IRH is also consistent with previous observations^2,21^. In addition, our results extend observation of Drew *et al*.^22^, showing that dementia can independently contribute to a higher risk of not only death but also IRH. Furthermore, we report the novel finding that after adjustment for relevant confounders, the presence of any infection event during advanced CKD was associated with a 30% higher risk of post-ESRD IRH.

Our findings for the short- and long-term negative impact of infection events during advanced CKD on post-ESRD outcome expand on the emerging theory that pre-ESRD conditions, such as blood pressure control^23^, cardiovascular medication nonadherence^24^, and abrupt decline in kidney function^25^, have a sustained impact on post-ESRD outcome. Our report adds to this literature by elucidating the impact of pre-dialysis infection. The aggravated negative impact of infection exposure we identified in the first year of dialysis therapy also supports the notion that incident dialysis patients experience the highest morbidity and mortality within the first year after starting dialysis^26^. This observation raises the possibility that infection events during advanced CKD directly affects clinical outcomes or that infection events are simply a surrogate marker for the severity of uremia that itself affects clinical outcomes. However, our study showed that the negative impact of infection exposure was not only restricted to the first year of dialysis but remained significant throughout the entire mean 3.6 years of follow-up period, indicating that uremia alone is not sufficient to interpret our whole results. Notably, Fig. 3 shows steeper slope in infection group was observed during the first to second year. Therefore, the negative impact of infection exposure may mainly impact on the early phase after dialysis initiation. Prior studies also demonstrated that rapid loss of kidney function before ESRD had an increased risk of death within the first year of ESRD^25,27^. As infection or other cause related AKI might confound our findings, we repeated our primary analysis excluding patients who had AKI during advanced CKD period and the effect estimates of the infection exposure were similar.

The underlying mechanisms linking infection events during advanced CKD to a higher risk of post-ESRD IRH or other adverse outcomes are likely to be multifactorial. In our study, the patients with advanced CKD who experienced at least one infection event had a higher prevalence of comorbidities. The progression of these comorbidities may conceivably serve as a link between pre-dialysis infection event and post-ESRD adverse outcomes. Alternatively, any infection event during advanced CKD may serve as a marker for patients with particularly poor control of these comorbidities. However, after adjustment for all the studied comorbidities, the risk of post-ESRD adverse outcomes was attenuated but remained statistically significant, supporting the idea that infections may trigger other risk factors, such as hypercatabolic state or chronic inflammation, leading to worse outcomes. Systemic inflammation has been shown to contribute to the link between infection and cardiovascular events^28,29^. Notably, this link is further supported by another finding of the current study, namely the independent association between pre-dialysis infection exposure and MACCE after dialysis initiation.

Another factor involved in the higher risk of post-ESRD adverse outcomes might be the result of insufficient handling of these advanced CKD patients after an infection event, potentially highlighting the importance of early intervention for these populations. Early referral to a nephrologist after any infection event in a patient with advanced CKD might be necessary because nephrologists are more likely to comprehensively manage worsening nutritional status, anemia, and volume status complicated by an infection event. Several observational studies have demonstrated that the quality of pre-dialysis CKD care independently predicts post-dialysis mortality^30,31^. Notably, our study found that a higher number of nephrologist visits during advanced CKD was associated with a lower risk of post-ESRD IRH, irrespective dialysis modality. Our finding that a higher risk of post-ESRD IRH is linked with pre-dialysis infection exposure among older patients and patients using tunneled-catheter is also supported by previous reports^19,21^, suggesting timely arteriovenous fistula creation. Moreover, incident dialysis patients with infection exposure or recurrent infections during advanced CKD appear particularly vulnerable to infections or other adverse events and may benefit from timely preventive strategies, such as nutrition resilience assessment^32^ and/or nutrition supplementation^33^, immunization against influenza and pneumococcus^34,35^, blood stream infection prevention collaborative interventions^36^, or appropriate management of comorbidities^37^. Further research is needed to confirm the beneficial effect of the aforementioned multifaceted interventions for such high-risk patients. Although this study provides novel information on the relationship between pre-dialysis infection exposure and post-ESRD adverse outcomes, they must be weighed against the potential limitations of the investigation. First, we excluded advanced CKD patients with a malignancy, an autoimmune disease, liver cirrhosis, or history of organ transplant because they may have had other factors linking infection and post-ESRD adverse outcomes. Therefore, our results may not be generalizable to the entire dialysis population. Second, our study design raises the possibility of selection bias as patients needed to survive those infection events and initiate dialysis to be included in the study. Third, we defined infection events by identifying only hospitalization and emergency department visits but some patients with a mild infection may have been treated in the outpatient setting, which led to underestimate the infection events. However, some disease can mimic a mild infection at initial presentation and those cases are difficult to ascertain due to either uncertain diagnostic accuracy or lack of definite information of infection type, potentially introducing misclassification bias. Fourth, our study was limited by the use of only principal discharge diagnoses to define infections; the true incidence of infection events may have been underestimated. We used diagnostic codes rather than validated clinical criteria to ascertain infection types, potentially resulting in some misclassifications. However, the primary objective of this study was to heighten awareness among clinicians regarding the significance of pre-dialysis infection events, like pre-dialysis cardiovascular disease, as potential risk factors for post-ESRD adverse outcomes and thus deserving of routine assessment. As such, misclassifications may not affect the relevance or accuracy of our results. Finally, although we adjusted for a large number of patient characteristics, we cannot rule out the possibility of residual confounding such as baseline nutritional status, exact eGFR at enrollment and speed of eGFR decline, or vaccination status. The observational nature of the study also makes it impossible to prove a causal link between the occurrence of infections and adverse outcome later on, meaning that the results should be regarded as hypothesis generating only and require further investigation.

In conclusion, the results of this study indicated that infection is a frequent event in patients with non-dialysis advanced CKD and that infection exposure during advanced CKD can lead to higher risks of post-ESRD adverse outcomes irrespective of all-cause mortality, IRH, or MACCE. Timely multifaceted preventive strategies among such high-risk incident dialysis patients may lead to improved outcomes and a lower burden on the health care system.

## Methods

### Ethics statement

This study was conducted in accordance with the Declaration of Helsinki. Because no identifiable personal information is included in the NHIRD, the Research Ethics Committee agreed that no informed consent was required. All experimental protocols were performed following the relevant guidelines and was approved by the Institutional Review Board of Chang Gung Memorial Hospital (IRB No.201701186B0).

### Data source

We analyzed longitudinal data from Taiwan’s National Health Insurance Research Database (NHIRD), which contains anonymized health care information collected prospectively from approximately 99.9% of Taiwan’s population. Only patients who had advanced CKD and then started permanent dialysis were included as the source population. Hence, we selected individuals who had a primary diagnosis of CKD and received erythropoiesis-stimulating agent (ESA) treatment. According to the reimbursement regulations of Taiwan’s National Health Insurance program, ESA is to be initiated when patients with CKD have an eGFR of 15 mL/min/1.73 m^2^or less and a hematocrit level below 28%. This definition of advanced CKD has been widely reported in previous NHIRD-based studies^38,39^. In addition, when patients with advanced CKD start permanent dialysis, they can receive a catastrophic illness certificate verified by the Bureau of National Health Insurance, exempting them from related copayments. Thus, the ESRD registry in the NHIRD is notably comprehensive and our selected cohort was highly representative of patients with advanced CKD who entered permanent dialysis.

### Exposure ascertainment

The primary exposure of interest was the number of infection episodes during pre-dialysis advanced CKD. An episode of infection was ascertained by identifying either hospitalizations or emergency department visits with a principal discharge diagnosis of selected infection-related International Classification of Diseases, Ninth Revision, Clinical Modification (ICD-9-CM) codes as indicated previously (Supplementary Table S3)^12^. To avoid double counting of the same infection, the infections recorded in emergency departments and those recorded for hospitalization were combined into one single infection episode if the admission date was within two weeks of the emergency visit date.

### Outcome and covariate assessment

The covariates we examined were age, sex, CKD duration, number of nephrologist outpatient visits in the previous year, dialysis modality, 11 baseline comorbidities, and 14 medications. Comorbidities were defined as at least 2 outpatient visits or one inpatient stay in the previous year. Most of the diagnostic codes used for these comorbidities have been validated in previous studies^40^. We obtained inpatient records only to ascertain history of heart failure, stroke, and myocardial infarction. Medications were identified by the filling of a prescription at least once or refilling a prescription for a chronic illness at least once within 3 months of the index date. The primary outcomes of interest were all-cause mortality and IRH after starting permanent dialysis. We identified first IRH event by using the same ICD-9-CM codes as for ascertaining infection exposure during advanced CKD. In addition to all-cause mortality, we also examined infection-related death by examining the cause of death in the main diagnosis in the discharge records for inpatient hospital deaths, the primary diagnosis of the last emergency visit, or hospitalization within 7 days of death for out-of-hospital deaths^41^. The secondary outcomes investigated were MACCE, comprising acute myocardial infarction, acute ischemic stroke, intracerebral hemorrhage, heart failure, and cardiovascular death, most of the diagnostic codes for which have been previously studied and validated^42,43^. The observation period was from the index date to death, first IRH or MACCE, or the end of the study, whichever occurred first. In addition, other events were analyzed, including sepsis death, catheter-related infection requiring catheter removal, readmission for any cause, and each MACCE component (Supplementary Tables S4 and S5). Death prior to the occurrence of IRH or MACCE was considered a competing risk event.

### Statistical analysis

We compared the continuous variables between groups (infection vs. non-infection) by using the independent sample t test. The categorical data between groups were compared using the chi-square test. The association between infection exposure prior to ESRD transition and risk of all-cause mortality was investigated using univariate and multivariable Cox proportional hazard models. The association between infection exposure and risk of other time to event outcomes (i.e., MACCE) was studied using a Fine and Gray subdistribution hazard model in which all-cause mortality during follow-up was a competing risk. Furthermore, to determine the robustness of our results and as a sensitivity analysis, we analyzed the risk of post-ESRD adverse outcomes according to four quartiles based on the annual number of infection episodes during pre-dialysis advanced CKD. We then examined linear trends by modeling the quartiles of these infection exposures as a continuous variable with and without adjustment for covariates. To compare the effect of pre-dialysis infection exposure on early and late post-ESRD adverse outcomes, the analyses were performed with the follow-up time restricted to the first year or with the entire follow-up period. The covariates adjusted for in the multivariable analyses were age, sex, CKD duration, number of nephrologist outpatient visits in the previous year, dialysis modality, 11 baseline comorbidities, 14 medications, and the index year (the year of dialysis initiation).

A sensitivity analysis was done by excluding patients who had AKI during advanced CKD because rapid decline of renal function caused by infection or other etiology might confound the primary analysis. At last, we also conducted subgroup analyses to examine whether the hazard of pre-dialysis infection exposure was consistent across different levels of the 4 pre-specified subgroups (sex, age, dialysis modality, and initial dialysis access type for hemodialysis). A two-sided P value of less than 0.05 was considered statistically significant and no adjustment for multiple testing (multiplicity) was made. All statistical analyses were performed using SAS version 9.4 (SAS Institute, Cary, NC), including the procedure ‘phreg’ for survival analysis and the macro ‘%cif’ for cumulative incidence functions.

## Supplementary information

Supplementary information.
