# Supplementary material for: Infection in Advanced Chronic Kidney Disease and Subsequent Adverse Outcomes after Dialysis Initiation: A Nationwide Cohort Study
Source: Sci Rep. 2020 Feb 19;10:2938. doi: 10.1038/s41598-020-59794-7 (PMC7031239; doi:10.1038/s41598-020-59794-7)
Supplement: Supplementary file 1 — Supplementary information. [file 41598_2020_59794_MOESM1_ESM.pdf]

Infection in Advanced Chronic Kidney Disease and Subsequent Adverse Outcomes after Dialysis Initiation: A Nationwide Cohort Study

Chih-Hsiang Chang [1,2], Pei-Chun Fan [1,2], George Kuo [1], Yu-Sheng Lin [3], Tsung-Yu Tsai [1,2], Su-Wei Chang [4,5], Ya-Chung Tian [1,2] Cheng-Chia Lee [1,2,*]

[1] Kidney research center, Chang Gung Memorial Hospital, Chang Gung University, College of medicine, Taoyuan, Taiwan

[2] Graduate Institute of Clinical Medical Sciences, College of medicine, Chang Gung University, Taoyuan, Taiwan

[3] Department of Cardiology, Chang Gung Memorial Hospital, Chiayi branch, Chiayi, Taiwan

[4] Clinical Informatics and Medical Statistics Research Center, Chang Gung University, Taoyuan, Taiwan

[5] Division of Allergy, Asthma, and Rheumatology, Department of Pediatrics, Chang Gung Memorial Hospital, Taoyuan, Taiwan

[*] Correspondence and reprint requests should be addressed to Cheng-Chia Lee, MD

E-mail: chia7181@gmail.com

Tel: +886-3-3281200-8181, Fax: +886-3-3282173

Supplementary Online Contents

Table S1. Detailed baseline characteristics of patients stratified by infection exposure status during pre-ESRD advanced CKD ------------------- 3

Table S2. Follow-up outcomes of primary interest in non-AKI patients with and without infection history during pre-dialysis advanced CKD-- 6

Table S3. ICD-9-CM code used in the current study --- 7

Table S4: Follow-up outcomes of secondary interest in patients with and without infection history during pre-dialysis advanced CKD --------10

Table S5: Follow-up outcomes of secondary interest stratified by annual number of infections during pre-dialysis advanced CKD -----------12

Figure S1. Cumulative incidence of post-ESRD IRH (A) and post-ESRD MACCE (B) in patients according to quartiles of annual number of infections during pre-dialysis advanced CKD -- 14

Supplementary Table S1. Detailed baseline characteristics of patients stratified by infection exposure status during advanced CKD

Variable	All (n = 62,872)	Infection (n = 20,566)	Non-infection (n = 42,306)	P value
Demographic				
Age (y)	63.1±13.7	65.6±13.6	61.9±13.6	<0.001
Age ≥ 65 y	29,836 (47.5)	11,393 (55.4)	18,443 (43.6)	<0.001
Female	30,404 (48.4)	11,322 (55.1)	19,082 (45.1)	
No. of nephrologist outpatient visits in the previous year	11.7±9.1	12.1±9.0	11.5±9.1	<0.001
Modality				<0.001
Hemodialysis	54,291 (86.4)	18,206 (88.5)	36,085 (85.3)	
Peritoneal dialysis	8,581 (13.6)	2,360 (11.5)	6,221 (14.7)	
Initial dialysis access type for hemodialysis				<0.001
Fistula	34,651 (63.8)	9,428 (51.8)	25,223 (69.9)	
Graft	6,155 (11.3)	2,602 (14.3)	3,553 (9.8)	
Tunneled-catheter	13,485 (24.8)	6,176 (33.9)	7,309 (20.3)	
Comorbidity				
Hypertension	56,987 (90.6)	18,699 (90.9)	38,288 (90.5)	0.090
Diabetes mellitus	36,952 (58.8)	12,489 (60.7)	24,463 (57.8)	<0.001
Ischemic heart disease	16,908 (26.9)	5,964 (29.0)	10,944 (25.9)	<0.001
Dementia	1,924 (3.1)	901 (4.4)	1,023 (2.4)	<0.001
History of heart failure	15,675 (24.9)	6,209 (30.2)	9,466 (22.4)	<0.001
Previous ischemic stroke	10,315 (16.4)	4,056 (19.7)	6,259 (14.8)	<0.001
Old myocardial infarction	4,189 (6.7)	1,658 (8.1)	2,531 (6.0)	<0.001
Chronic obstructive pulmonary disease	4,257 (6.8)	1,778 (8.6)	2,479 (5.9)	<0.001
Peripheral arterial disease	2,257 (3.6)	886 (4.3)	1,371 (3.2)	<0.001

Variable	All (n = 62,872)	Infection (n = 20,566)	Non-infection (n = 42,306)	P value
Previous hemorrhage stroke	1,523 (2.4)	630 (3.1)	893 (2.1)	<0.001
Polycystic kidney disease	1,405 (2.2)	478 (2.3)	927 (2.2)	0.290
Hypoalbuminemia	2,794 (4.4)	1,754 (8.5)	1,040 (2.5)	<0.001
Primary renal disease				<0.001
DM nephropathy	23,671 (37.6)	7,940 (38.6)	15,731 (37.2)	
Chronic glomerulonephritis	25,784 (41.0)	8,257 (40.1)	17,527 (41.4)	
HTN nephropathy	3,011 (4.8)	1,077 (5.2)	1,934 (4.6)	
Polycystic kidney	1,405 (2.2)	478 (2.3)	927 (2.2)	
Interstitial nephritis	1,142 (1.8)	356 (1.7)	786 (1.9)	
Obstructive nephropathy	924 (1.5)	441 (2.1)	483 (1.1)	
Others	6,935 (11.0)	2,017 (9.8)	4,918 (11.6)	
Medications				
Steroid	3,965 (6.3)	1,520 (7.4)	2,445 (5.8)	<0.001
Other immunosuppressive agents	580 (0.9)	178 (0.9)	402 (1.0)	0.297
Antiplatelet	21,514 (34.2)	7,287 (35.4)	14,227 (33.6)	<0.001
ACEI/ARB	33,522 (53.3)	10,521 (51.2)	23,001 (54.4)	<0.001
Antihypertensive agents	54,811 (87.2)	17,539 (85.3)	37,272 (88.1)	<0.001
Loop diuretics	43,238 (68.8)	14,389 (70.0)	28,849 (68.2)	<0.001
K-sparing diuretics	2,337 (3.7)	800 (3.9)	1,537 (3.6)	0.110
Oral hypoglycemic agent	22,319 (35.5)	7,367 (35.8)	14,952 (35.3)	0.239
Insulin	18,384 (29.2)	6,644 (32.3)	11,740 (27.8)	<0.001
Proton pump inhibitor	12,291 (19.5)	5,138 (25.0)	7,153 (16.9)	<0.001
NSAID (including COX2)	7,569 (12.0)	2,587 (12.6)	4,982 (11.8)	0.004

Variable	All (n = 62,872)	Infection (n = 20,566)	Non-infection (n = 42,306)	P value
Statin	16,496 (26.2)	4,809 (23.4)	11,687 (27.6)	<0.001
Fibrate or Gemfibrozil	2,793 (4.4)	829 (4.0)	1,964 (4.6)	<0.001
Vitamin D therapy	8,170 (13.0)	2,332 (11.3)	5,838 (13.8)	<0.001
Follow-up duration (y)	3.6±2.6	3.2±2.4	3.8±2.6	<0.001

Abbreviations: ACEI, angiotensin converting enzyme inhibitor; ARB, angiotensin receptor blocker; CKD, chronic kidney disease; COX-2, cyclo-oxygenase-2 inhibitor; NSAID, non-steroidal anti-inflammatory drug.

Data are presented as frequency (percentage) or mean ± standard deviation.

Supplementary Table S2. Follow-up outcomes of primary interest in non-AKI patients with and without infection history during pre-dialysis advanced CKD (the sensitivity analysis)

	No. of events (%)			Infection vs. non-infection[b]			
	All	Infection	Non-infection	Univariate		Multivariable[c]	
Outcome	(n = 49,836)	(n = 15,033)	(n = 34,803)	HR (95% CI)	P value	HR (95% CI)	P value
1-year follow-up							
All-cause mortality	3,863 (7.8)	1,703 (11.3)	2,160 (6.2)	1.88 (1.77, 2.00)	<0.001	1.31 (1.23, 1.41)	<0.001
IRH	12,530 (25.1)	5,167 (34.4)	7,363 (21.2)	1.79 (1.73, 1.85)	<0.001	1.45 (1.39, 1.50)	<0.001
Infection death	2,001 (4.0)	1,002 (6.7)	999 (2.9)	2.37 (2.17, 2.59)	<0.001	1.52 (1.39, 1.67)	<0.001
MACCE[a]	4,603 (9.2)	1,764 (11.7)	2,839 (8.2)	1.47 (1.38, 1.55)	<0.001	1.14 (1.08, 1.22)	<0.001
At the end of follow-up							
All-cause mortality	19,379 (38.9)	6,774 (45.1)	12,605 (36.2)	1.47 (1.43, 1.51)	<0.001	1.18 (1.15, 1.22)	<0.001
IRH	26,742 (53.7)	9,164 (61.0)	17,578 (50.5)	1.46 (1.43, 1.50)	<0.001	1.28 (1.25, 1.32)	<0.001
Infection death	8,790 (17.6)	3,358 (22.3)	5,432 (15.6)	1.57 (1.50, 1.63)	<0.001	1.25 (1.19, 1.31)	<0.001
MACCE[a]	15,684 (31.5)	5,165 (34.4)	10,519 (30.2)	1.22 (1.18, 1.26)	<0.001	1.06 (1.03, 1.10)	<0.001

Abbreviations: CI, confidence interval; CKD, chronic kidney disease; HR, hazard ratio; IRH, infection-related hospitalization; MACCE, major adverse cardiac and cerebrovascular event.

[a] Any patient with acute myocardial infarction, acute ischemic stroke, intracerebral hemorrhage, heart failure, or cardiovascular death.

[b] Except for all-cause mortality, the outcomes were estimated using a subdistribution hazard model that considered all-cause mortality a competing risk.

[c] Adjusted for the variables listed in Supplementary Table S1 except primary renal disease; the follow-up duration was replaced with the index year.

Supplementary Table S3. ICD-9-CM code used in the current study

Variable	Code
Dialysis	585.xx (Catastrophic illness certificate)
Infection	0031, 0362, 0380, 03810, 03811, 03812, 03819, 0382, 0383, 03840, 03841, 03842, 03843, 03844, 03849, 0388, 0389, 04082, 0545, 1125, 78552, 7907, 7908, 99591, 99592, 03282, 03640, 03641, 03642, 03643, 07420, 07421, 07422, 07423, 11281, 11503, 11504, 11593, 11594, 1303, 3910, 3911, 3912, 3918, 3919, 3920, 4210, 4211, 4219, 4220, 42292, 00321, 0360, 0361, 0470, 0471, 0478, 0479, 048, 0490, 0491, 0498, 0499, 0530, 05310, 05314, 0543, 05472, 05474, 0550, 05600, 05601, 05609, 05821, 05829, 0621, 0622, 0623, 0625, 0628, 0629, 0638, 0639, 064, 06641, 06642, 0721, 0722, 11283, 1142, 11501, 11591, 1300, 3200, 3201, 3202, 3203, 3207, 32081, 32082, 32089, 3209, 3210, 3211, 3212, 3230, 32301, 32302, 3231, 3234, 32341, 32342, 3240, 3241, 3249, 03283, 5670, 5671, 5672, 56721, 56722, 56723, 56729, 56789, 5679, 0030, 0038, 0039, 0040, 0041, 0043, 0048, 0049, 0050, 0051, 0052, 0053, 0054, 00581, 00589, 0059, 0071, 0074, 0075, 00800, 00801, 00802, 00803, 00804, 00809, 0081, 0082, 0083, 00841, 00842, 00843, 00844, 00845, 00846, 00847, 00849, 0085, 00861, 00862, 00863, 00864, 00865, 00866, 00867, 00869, 0088, 0090, 0091, 0092, 0093, 0392, 0700, 0701, 07043, 07053, 0723, 07271, 11285, 1305, 5400, 5401, 5409, 541, 542, 56201, 56203, 56211, 56213, 566, 56781, 5695, 5720, 5721, 5750, 57510, 03284, 0720, 59010, 59011, 5902, 5903, 59080, 59081, 5909, 5950, 5954, 59589, 5959, 5970, 59800, 59801, 5990, 6010, 6012, 6013, 6014, 6019, 6031, 6040, 60490, 60491, 6071, 6072, 6080, 6084, 6140, 6142, 6143, 6145, 6150, 6159, 6163, 6164, 00322, 01100, 01101, 01102, 01103, 01104, 01105, 01106, 01110, 01111, 01112, 01113, 01114, 01115, 01116, 01120, 01121, 01122, 01123, 01124, 01125, 01126, 01130, 01131, 01132, 01133, 01134, 01135, 01136, 01150, 01151, 01152, 01153, 01154, 01155, 01156, 01160, 01161, 01162, 01163, 01164, 01165, 01166, 01170, 01171, 01172, 01173, 01174, 01175, 01176, 01180, 01181, 01182, 01183, 01184, 01185, 01186, 01190, 01191, 01192, 01193, 01194, 01195, 01196, 0310, 0330, 0338, 0339, 0391, 0521, 0551, 0730, 0796, 1124, 1140, 1145, 11505, 11595, 1304, 1363, 4650, 4658, 4659, 4660, 46611, 46619, 4800, 4801, 4802, 4803, 4808, 4809, 481, 4820, 4821, 4822, 48230, 48231, 48232, 48239, 48240, 48241, 48242, 48249, 48281, 48282, 48283, 48284, 48289, 4829, 4830, 4831, 4838, 4841, 4843, 4846, 4847, 4848, 485, 486, 4870,

Variable	Code
	4871, 488, 4880, 4881, 490, 49122, 4941, 5100, 5109, 5111, 5130, 5131, 5192, 0311, 03285, 0390, 0400, 37601, 6800, 6801, 6802, 6803, 6804, 6805, 6806, 6807, 6808, 6809, 68100, 68101, 68110, 68111, 6819, 6820, 6821, 6822, 6823, 6824, 6825, 6826, 6827, 6828, 6829, 684, 6850, 6868, 6869, 72886, 9101, 9103, 9109, 9111, 9113, 9119, 9121, 9123, 9129, 9131, 9133, 9139, 9141, 9143, 9149, 9151, 9153, 9159, 9161, 9163, 9169, 9171, 9173, 9179, 9191, 9193, 9199, 00323, 00324, 03682, 37603, 05671, 71100, 71101, 71102, 71103, 71104, 71105, 71106, 71107, 71108, 71109, 71140, 71141, 71142, 71143, 71144, 71145, 71146, 71147, 71148, 71149, 71150, 71151, 71152, 71153, 71154, 71155, 71156, 71157, 71158, 71159, 71160, 71161, 71162, 71163, 71164, 71165, 71166, 71167, 71168, 71169, 71180, 71181, 71182, 71183, 71184, 71185, 71186, 71187, 71188, 71189, 71190, 71191, 71192, 71193, 71194, 71195, 71196, 71197, 71198, 71199, 73000, 73001, 73002, 73003, 73004, 73005, 73006, 73007, 73008, 73009, 73020, 73021, 73022, 73023, 73024, 73025, 73026, 73027, 73028, 73029, 73080, 73081, 73082, 73083, 73084, 73085, 73086, 73087, 73088, 73089, 73090, 73091, 73092, 73093, 73094, 73095, 73096, 73097, 73098, 73099, 99662, 99931, 99668, 53086, 53641, 56961, 99660, 99661, 99663, 99664, 99665, 99666, 99667, 99669, 99731, 99802, 99851, 99859, 9993, 99939
Organ transplant	V42.0, V42.1, V42.6, V42.7, V42.8x, 996.81, 996.82, 996.83, 996.84, 996.85 (Catastrophic illness certificate)
Malignancy	140.xx－208.xx (Catastrophic illness certificate)
Liver cirrhosis	571.2, 571.5, 571.6 (Catastrophic illness certificate)
Autoimmune disease	710.0, 710.1, 714.0, 710.4, 710.3, 446.0, 446.2, 446.4, 446.5, 443.1, 446.7, 136.1, 694.4, 710.2, 555.xx, 556.xx, 714.30－714.33 (Catastrophic illness certificate)
Chronic kidney disease	580.xx–589.xx, 403.xx–404.xx, 016.0x, 095.4x, 236.9x, 250.4x, 274.1x, 442.1x, 447.3x, 440.1x, 572.4x, 642.1x, 646.2x, 753.1x, 283.11, 403.01, 404.02, 446.21
Polycystic kidney disease	753.12, 753.13, 753.14
Hypertension	401.xx–405.xx
Diabetes mellitus	250.xx
Chronic obstructive pulmonary disease	491.xx, 492.xx, 496.xx

Variable	Code
Peripheral arterial disease	440.0x, 440.2x, 440.3x, 440.8x, 440.9x, 443.xx, 444.0x, 444.22, 444.8x, 447.8x, 447.9x
Ischemic heart disease	410.xx–414.xx
Dementia	290.xx, 294.xx
Heart failure	428.xx
Ischemic stroke	433.xx–434.xx
Hemorrhage stroke	430.xx–432.xx
Old myocardial infarction	410.xx, 412.xx
Sepsis	038.xx, 790.7
Acute myocardial infarction	410.xx
Acute ischemic stroke	433.xx-436.xx, excluded 433.00, 433.10, 433.20, 433.30, 433.80, 433.90, 434.90, 434.00, 434.10, and 434.90
Intracerebral hemorrhage	431.xx
Cardiovascular death	390.xx – 459.xx

ICD-9-CM, International Classification of Diseases, Ninth Revision, Clinical Modification.

Supplementary Table S4. Follow-up outcomes of secondary interest in patients with and without infection history during pre-dialysis advanced CKD

	Number of event (%)			Infection vs. Non-infection#			
	All	Infection	Non-infection	Univariate		Multivariable‡	
**Outcome**	**(n = 62,872)**	**(n = 20,566)**	**(n = 42,306)**	**HR (95% CI)**	**P value**	**HR (95% CI)**	**P value**
1 year follow-up							
Sepsis death	2,076 (3.3)	1,149 (5.6)	927 (2.2)	2.60 (2.38, 2.83)	<0.001	1.61 (1.47, 1.77)	<0.001
Catheter-related infection§	5,996 (9.5)	2,556 (12.4)	3,440 (8.1)	1.56 (1.48, 1.64)	<0.001	1.19 (1.13, 1.26)	<0.001
Readmission	32,697 (52.0)	12,239 (59.5)	20,458 (48.4)	1.38 (1.35, 1.42)	<0.001	1.19 (1.16, 1.21)	<0.001
Acute myocardial infarction	969 (1.5)	355 (1.7)	614 (1.5)	1.19 (1.05, 1.36)	0.009	1.04 (0.90, 1.19)	0.622
Acute ischemic stroke	1,523 (2.4)	510 (2.5)	1,013 (2.4)	1.04 (0.93, 1.15)	0.518	0.91 (0.82, 1.02)	0.110
Intracerebral hemorrhage	426 (0.7)	141 (0.7)	285 (0.7)	1.02 (0.83, 1.25)	0.867	1.01 (0.81, 1.24)	0.955
Heart failure	1,392 (2.2)	539 (2.6)	853 (2.0)	1.30 (1.17, 1.45)	<0.001	1.03 (0.92, 1.16)	0.560
Cardiovascular death	3,062 (4.9)	1,484 (7.2)	1,578 (3.7)	1.98 (1.84, 2.12)	<0.001	1.36 (1.26, 1.47)	<0.001
At the end of follow-up							
Sepsis death	8,068 (12.8)	3,392 (16.5)	4,676 (11.1)	1.62 (1.55, 1.69)	<0.001	1.27 (1.21, 1.33)	<0.001
Catheter-related infection§	12,293 (19.6)	4,686 (22.8)	7,607 (18.0)	1.35 (1.30, 1.40)	<0.001	1.14 (1.10, 1.19)	<0.001
Readmission	50,137 (79.7)	16,970 (82.5)	33,167 (78.4)	1.27 (1.25, 1.29)	<0.001	1.13 (1.11, 1.15)	<0.001
Acute myocardial infarction	3,487 (5.5)	1,116 (5.4)	2,371 (5.6)	0.99 (0.93, 1.07)	0.860	0.99 (0.91, 1.06)	0.695
Acute ischemic stroke	4,806 (7.6)	1,528 (7.4)	3,278 (7.7)	0.98 (0.92, 1.04)	0.519	0.95 (0.89, 1.01)	0.127
Intracerebral hemorrhage	1,596 (2.5)	469 (2.3)	1,127 (2.7)	0.87 (0.79, 0.97)	0.015	0.95 (0.85, 1.06)	0.379
Heart failure	4,045 (6.4)	1,406 (6.8)	2,639 (6.2)	1.13 (1.06, 1.20)	<0.001	1.02 (0.95, 1.09)	0.535

Outcome	Number of event (%)			Infection vs. Non-infection#			
	All	Infection	Non-infection	Univariate		Multivariable‡	
	(n = 62,872)	(n = 20,566)	(n = 42,306)	HR (95% CI)	P value	HR (95% CI)	P value
Cardiovascular death	13,072 (20.8)	4,871 (23.7)	8,201 (19.4)	1.33 (1.29, 1.38)	<0.001	1.11 (1.06, 1.15)	<0.001

CKD, chronic kidney disease; HR, hazard ratio; CI, confidence interval;

§ Catheter-related infection requiring catheter removal

# Outcomes were estimated using subdistribution hazard model which considered all-cause mortality as a competing risk;

‡ Adjusted for variables listed in Table S1 except primary renal disease, the follow up duration was replaced with index year.

Supplementary Table S5. Follow-up outcomes of secondary interest stratified by annual number of infections during pre-dialysis advanced CKD

	No. of event (%), categorized by annual number of previous infections					
**Outcome#**	**Group 1 (n = 5,142)**	**Group 2 (n = 5,154)**	**Group 3 (n = 5,122)**	**Group 4 (n = 5,148)**	**P trend**	**P trend‡**
1 year follow-up						
Sepsis death	153 (3.0)	219 (4.2)	330 (6.4)	447 (8.7)	<0.001	<0.001
Catheter-related infection§	496 (9.6)	577 (11.2)	670 (13.1)	813 (15.8)	<0.001	<0.001
Readmission	2,562 (49.8)	3,014 (58.5)	3,264 (63.7)	3,399 (66.0)	<0.001	<0.001
Acute myocardial infarction	59 (1.1)	99 (1.9)	98 (1.9)	99 (1.9)	0.003	0.550
Acute ischemic stroke	111 (2.2)	126 (2.4)	136 (2.7)	137 (2.7)	0.080	0.267
Intracerebral hemorrhage	31 (0.6)	31 (0.6)	38 (0.7)	41 (0.8)	0.173	0.906
Heart failure	85 (1.7)	126 (2.4)	148 (2.9)	180 (3.5)	<0.001	0.005
Cardiovascular death	214 (4.2)	325 (6.3)	425 (8.3)	520 (10.1)	<0.001	<0.001
At the end of follow-up						
Sepsis death	669 (13.0)	757 (14.7)	903 (17.6)	1,063 (20.6)	<0.001	<0.001
Catheter-related infection§	1,045 (20.3)	1,113 (21.6)	1,218 (23.8)	1,310 (25.4)	<0.001	<0.001
Readmission	4,011 (78.0)	4,229 (82.1)	4,307 (84.1)	4,423 (85.9)	<0.001	<0.001
Acute myocardial infarction	222 (4.3)	292 (5.7)	298 (5.8)	304 (5.9)	0.002	0.986
Acute ischemic stroke	342 (6.7)	392 (7.6)	402 (7.8)	392 (7.6)	0.129	0.519
Intracerebral hemorrhage	108 (2.1)	119 (2.3)	122 (2.4)	120 (2.3)	0.576	0.302
Heart failure	280 (5.4)	363 (7.0)	372 (7.3)	391 (7.6)	<0.001	0.192
Cardiovascular death	954 (18.6)	1,216 (23.6)	1,255 (24.5)	1,446 (28.1)	<0.001	0.002

CKD, chronic kidney disease;

The median number of previous infection event was 0.7, 1.8, 4.1 and 12.2 in group 1, group 2, group 3 and group 4 respectively;

§ Catheter-related infection requiring catheter removal

# Outcomes were estimated using subdistribution hazard model which considered all-cause mortality as a competing risk;

‡ Adjusted for variables listed in Table S1 except primary renal disease, the follow up duration was replaced with index year.

Supplementary Fig S1. Cumulative incidence of post-ESRD IRH (a) and post-ESRD MACCE (b) in patients according to quartiles of annual number of infections during pre-dialysis advanced CKD.

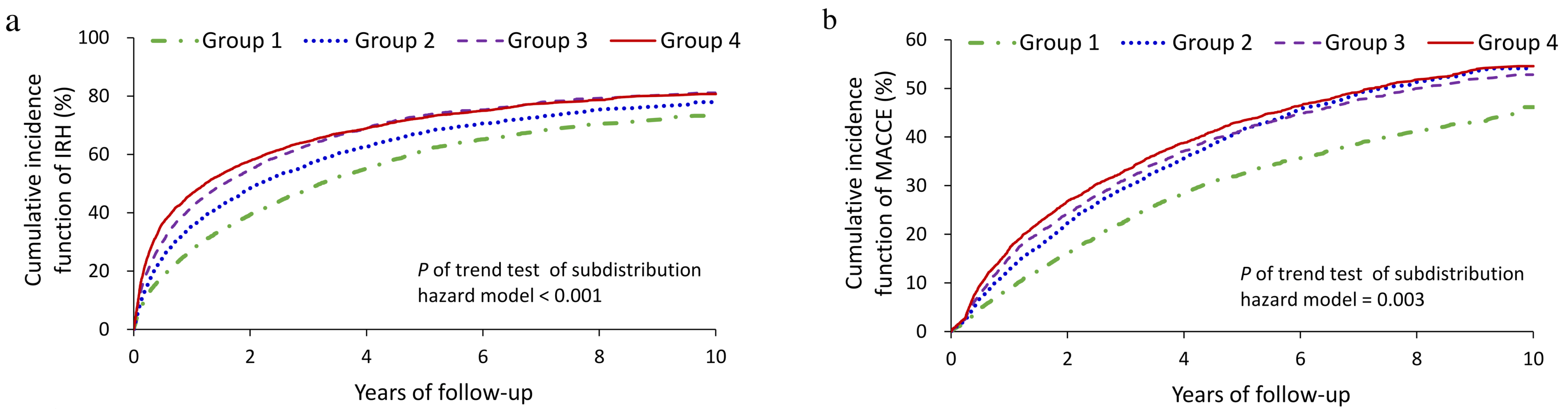

(a) No. at risk:

Group 1	5142	2262	1058	462	160	0
Group 2	5154	1837	771	310	97	0
Group 3	5122	1588	623	269	89	0
Group 4	5148	1525	689	303	99	1

(b) No. at risk:

Group 1	5142	3094	1653	846	324	1
Group 2	5154	2826	1408	625	214	0
Group 3	5122	2583	1220	535	182	0
Group 4	5148	2540	1302	605	231	1

CKD, chronic kidney disease; ESRD, end stage renal disease; IRH, infection-related hospitalization; MACCE, major adverse cardiac and cerebrovascular event
